# The impact of community-based health insurance on health-related quality of life and associated factors in Ethiopia: a comparative cross-sectional study

**DOI:** 10.1186/s12955-018-0946-3

**Published:** 2018-05-31

**Authors:** Teklemichael Gebru, Kifle Lentiro

**Affiliations:** Department of Public Health, College of Medicine and Health Science, Wolkite University, Addis Ababa, Ethiopia

**Keywords:** Household head, Health insurance, Quality of life

## Abstract

**Background:**

Quality of life can be used to measure the effect of intervention on health related conditions. Health insurance contributes positive effect on availability of medical supplies and empowerment of women and children on financial healthcare. Therefore, the study was aimed to measure the impact of Community-Based Health Insurance on HRQoL and associated socio-demographic factors.

**Methods:**

A comparative community based cross-sectional study was employed. Data was collected by trained enumerators using World Health Organization QoL-BREF tool from a sample of 1964 (982 CBHI insured and 982 un-insured) household heads selected by probability proportional to size. A descriptive summery, simple and multiple linear regression analysis was applied to describe the functional predictors of HRQoL. The study was ethically approved by IRB of Wolkite University.

**Results:**

The HRQoL score among CBHI insured family heads was 63.02 and 58.92 for un-insured family heads. The overall variation in HRQoL was explained due to; separated marital condition which reduced the HRQoL by 4.30% than those living together [^β^ = − 0.044, 95% CI (− 5.67, − 0.10)], daily laborer decreased HRQoL by 7.50% [^β^ **= −** 0.078, 95% CI (− 12.91, − 4.10)], but employment increased by 5.65% than farmers [^β^ **=** 0.055, 95% CI (2.58, 17.59)]. QoL increased by 6.4 and 6.93% among primary and secondary level educated household heads than those household heads who could not read and write [^β^ **=** 0.062, 95% CI (0.75, 4.31)] and [^β^ **=** 0.067, 95% CI (1.84, 7.99)], respectively. As family size increased by one households’ head, HRQoL decreased by 18.21% [^β^ = − 0.201, 95% CI (− 2.55, − 1.63)], as wealth index increased by one unit, HRQoL decreased by 32.90% [^β^ = − 0.306, 95% CI (− 5.15, − 3.86)] and QoL among CBHI insured household heads increased by 12.41% than those un-insured family heads [^β^ = 0.117, 95% CI (2.98, 6.16)].

**Conclusions:**

The study revealed that significant difference in quality of life was found among the two groups; health insurance had positive effect on quality of life. Triggered, the government shall expand the scheme into other similar areas’ and further efforts should be made on the scheme service satisfaction to ensure its continuity.

## Background

In terms health related indicators; Ethiopia ranks low even as compared to other low income countries [1]. The country bears high burden of preventable communicable diseases. According to the country ministry of health (MoH) 2015 report, the top leading causes of mortality were malaria, pneumonia and respiratory tract diseases [2, 3]. In spite to this high burden, utilization of modern health care services is limited [3]. One of the reasons for low utilization of healthcare services is the direct user-fee charges [4].

In Ethiopia, 38.5% of the total health expenditure was covered through out-of-pocket charges, which is higher than that of other African countries, which was 30.6% in 2008 [3, 5]. Nevertheless, Ethiopia’s per capita public spending for health (14 US$ in 2008) remains far below even that of other African and low income countries (83 US$ and 32 US$, respectively in 2008) [5].

Health care expenses are devastating and have long term effect on economic situations to majority of household heads in Ethiopia. Consequently, it was suggested that alternative mechanisms such as health taxes should be established to cover health care expenses [6]. Moving away from out of pocket charges for healthcare at the time of use is an important step towards in averting the financial hardship related with paying for health service [7]. But, in 2008 the government planned to cover the prepaid only 1.5% of the total private expenditure on health in Ethiopia [5].

To increase the prepaid plan coverage and access to modern health care services, the Ethiopian government has introduced Community Based Health Insurance (CBHI). The scheme was piloted since 2011 in 13 districts with the objective to draw a lesson for scale-up at countrywide level. Currently the scheme covers 202 districts, including 52 from South Nation Nationality People’s Region (SNNPR). Increased and improved cash flow has had a positive effect on the availability of medications and other supplies, which turn to improve the quality of health services. However, there is no quantified evidence whether the CBHI may contribute to Health-Related Quality of Life (HRQoL) or yet not. Therefore, the purpose of this study was to assess the impact of CBHI on health-related quality of life. It was believed that this study will helps to provide evidence based decisions by policy makers to benefit the community of the country and beyond.

## Methods

### Study area and period

The study was conducted in SNNPR which is one of the largest regions in Ethiopia, covers for more than 10% of the country’s land area with an estimated 112,343.19 km^2^. Based on 2016 point estimate, the region has a population of 15,927,649, including 7,916,042 men and 8,011,607 women. The region was administratively divided into 13 zones, 133 Woredas (administrative levels higher than kebeles) and 3512 Kebeles (the smallest administrative level in the country**)**. Healthcare service of the region was renders through 45 Hospitals, 248 Health Centers and 3729 Health Posts. The study was conducted in Dale Woreda (Yirgalem) as a best pilot CBHI implementer since 2011 and non-CBHI covered Gorche Woreda which has similar socio-demographic character with the piloted Woreda (Yirgalem) [8]. The study was employed in February, 2017.

### Study design and population

A community based comparative cross-sectional study was employed. Source populations of the study were all household heads whereas households heads found in randomly sampled household heads’ were study population. Household heads reside at least for six months in the study area were included in the study however household heads’ who were included in to the scheme within six months of the study were excluded to minimize immature effect of the scheme.

### Sample size determination and procedure

Sample size was calculated using STATCALC program of EPI INFO version 7 statistical packages for windows by assuming; mean score 2.4 (± 0.8) difference of HRQoL (as outcome variable), 95% confidence interval (Zα_/2_), 80% power, insured and un-insured household head ratio of 1:1, and 10% expected non-response rate [8]. Accordingly, the required sample size was 1967 household heads. The best CBHI pilot implementation performer of the country Dale Woreda was selected as CBHI covered Woreda and Gorche Woreda as non-CBHI covered Woreda which has similar socio demographic character was selected, in these two Woredas there were 36 and 22 Kebeles, and 48,971 and 23,705 households, respectively. Sampling frame was prepared with cumulative frequency for each district [8]. Then independently for each district probability proportional to size sampling method was used to select the sampling unit household. Accordingly, we plan to collect data and distributed a questionnaire of 1967 that consists socio-demographic and HRQoL related issues.

### Data collection and quality assurance

An adapted World Health Organization Quality of Life Biomedical Research and Educational Facility (WHOQoL-BREF) data collection tool was used in this study. First, the tool was adopted in English then translated into Amharic and finally back translated in to English by another expert to keep its consistency. The tool consists two items on overall general health and 24 items divided into four domains; 7 items physical health, 6 items psychological health, 3 items social relationships and 8 items environmental health rated on five-point likert scale [9]. Twenty health extension worker data collectors and four BSc holder public health supervisors were involved in the data collection process. The overall data collection process was coordinated by researchers. Moreover, the questioner was pre-tested on 5% of the actual sample size in the area with population having similar socio-demographic status with study population.

Training was given to data collectors prior to the start of data collection process for three-day. Lecture, mock interview and field practice were included in the training process. Even through, supervisors were trained together with the data collectors; orientation was given separately on how to supervise the data collectors. Moreover, daily based check-up on 10% of the filled questionnaire each day were made and incomplete questionnaire was referred back for completion and data was validated for similarity through double data entry by Epi Data 3.1 statistical software for windows.

### Data processing and analysis

The collected data was checked for completeness, edited, coded, entered to Epi-data 3.1 software and cleaned. Then for analysis it was export to Statistical Package for Social Science (SPSS) version 20.0 for windows. Descriptive summary was calculated for socio-demographic characteristics such as mean and proportions. Based on the WHOQoL-BREF guideline after inversely coding negatively coded items raw domain score was computed and transformed the total HRQoL score. Moreover, Levene’s test was used to assess homogeneity of the two populations (insured and un-insured) and principal component analysis was employed for wealth index. Cronbach’s alpha coefficient with 0.70 and above was accepted.

Simple linear regression was applied to see the association between factors and HRQoL as a first phase screening. To avoid unstable estimate variables with *p*-value = < 0.25 were candidate for the final regression model [10]. Then, multiple leaner regression was applied in order to control the effect of confounding factors and to describe the functional association between the socio-demographic factors and the total score HRQoL. Beta was determined to estimate the strength of association with 95% confidence interval (CI). For all statistical significance tests, the cut- off value set is *p* <  0.05.

## Results

### Socio-demographic characteristics

We planned to participate 1967 household heads, however, 1955 were included in the study; this makes 99.44% response rate. Among the study participants 1318 (67.4%) were husband (male) respondents. The mean age of study participants were 40 with standard deviation of 11 years. Of the respondents 1234 (72.1%) were farmers by their jobs. More than half of the study participant 1121 (57.3%) could not read & write. On the other hand, among the interviewed household heads, 1031 (52.7%) have greater than five family size. Moreover, by their socio-demographic characteristics, the two populations were statistically homogenous only by educational status and marital condition whereas they were statistically different by age, gender, family size and wealth index (Table 1).

**Table 1:** Socio-demographic characteristics of study participants in SNNPR, *n* = 1955, February, 2017

Variable	Household condition on CBHI				Homogeneity test	
	Insured		Un-insured			
	Count (*n*)	Percent (%)	Count (*n*)	Percent (%)	Chi-Square	*P*-value
Respondent type					17.74	< 0.00
Husband	613	62.9	705	71.9		
Wife	361	37.1	276	28.1		
Age in year (mean, SD)	41(± 10)		40(± 11)		531.34	< 0.00
Current job					40.59	< 0.00
House wife	355	36.4	264	26.9		
Farmer	556	57.1	678	69.1		
Laborer	35	3.6	30	3.1		
Employed	28	2.9	9	0.9		
Marital condition					0.32	0.57
Live together	872	89.5	863	88.0		
Separate/divorce/widow	102	10.4	118	12.0		
Educational level					6.34	0.09
Could not read & write	564	57.9	557	56.8		
Primary	332	34.1	344	35.1		
Secondary and above	78	8.0	74	8.1		
Family size					99.95	< 0.00
Less than or equal to 5	350	35.9	574	58.5		
Greater than 5	624	64.1	407	41.5		
Wealth index (quintile)					48.57	< 0.00
Poorest	149	15.30	129	13.1		
Poor	244	25.1	204	20.8		
Medium	244	26.1	176	17.9		
Rich	166	17.0	270	27.5		
Richest	161	16.5	202	20.6		

### Health related quality of life score

Among the two group of the population, the highest mean and percentage satisfaction of HRQoL for insured family were revealed psychological domain (mean = 31.12 (± 5.65); percentage = 86.13) and un-insured were physical health domain (mean = 28.63 (± 6.76); percentage = 66.54) and the lowest satisfaction of HRQoL for both group of the population were social relationship domain (mean = 8.86 (± 2.94); percentage = 48.79) and (mean = 8.67 (± 3.62); percentage = 60.75), respectively. From the Levene’s test for homogeneity, the two populations were statistically different by physical health and Psychological domains (F _(1, 1953)_ = 128.95.77, *p* = < 0.00) and (F _(1, 1953)_ = 309.61, *p* = < 0.00), respectively. The transformed total HRQoL score among CBHI insured family heads was 63.02 and 58.92 for un-insured family and the two populations were statistically different (F _(1, 1953)_ = 21.77, *p* = < 0.00) (Table 2).

**Table 2:** Quality of life score among insured and un-insured household head study participants in SNNPR, *n* = 1955, February, 2017

Variables	Household condition on CBHI				Test of Homogeneity	
	Insured		Un-insured			
	Mean (±SD)	Transformed score	Mean (±SD)	Transformed score	F	*P*-value
Physical health	22.17(± 6.72)	54.17	28.63(± 6.76	66.54	128.95	< 0.00
Psychological	31.12(± 5.65)	86.13	24.01(± 11.28)	47.24	309.61	< 0.00
Social relationship	8.86(± 2.94)	48.79	8.67(± 3.62)	60.75	1.56	0.21
Environment	29.15(± 6.39)	62.97	25.57(± 8.54)	61.14	2.94	0.09
Quality of life		63.02		58.92	21.77	< 0.00

### Factors associated with health related quality of life

The overall variation in HRQoL was contributed due to; separated marital condition was reduced the HRQoL by 4.30% than those living together [^β^ = − 0.044, 95% CI (− 5.67, − 0.10)], daily laborer household heads decreased HRQoL by 7.50% [^β^ **= −** 0.078, 95% CI (− 12.91, − 4.10)], but employment increased by 5.65% than those who were farmers [^β^ **=** 0.055, 95% CI (2.58, 17.59)]. HRQoL among primary and secondary educated household heads increased by 6.4 and 6.93% than those household heads who could not read and write [^β^ **=** 0.062, 95% CI (0.75, 4.31)] and [^β^ **=** 0.067, 95% CI (1.84, 7.99)], respectively. As family size increased by one households’ head, HRQoL decreased by 18.21% [^β^ = − 0.201, 95% CI: (− 2.55, − 1.63)]. Moreover, as wealth index increased by one unit, HRQoL decreased by 32.90% [^β^ = − 0.306, 95% CI (− 5.15, − 3.86)] and HRQoL among CBHI insured household heads were increased by 12.41% than those un-insured family heads [^β^ = 0.117, 95% CI (2.98, 6.16)] (Table 3).

**Table 3:** Predictors to quality of life among household head study participants in SNNPR, *n* = 1955, February, 2017

Variable	Quality of life		
	Beta with 95% CI		
	Unstandardized	Standardized	*P*-value
Age in year	−0.041(− 0.16, 0.01)	− 0.038 (− 0.15, 0.01)	0.069
Marital condition			
Live together	–	–	–
Separate/divorce/widow	− 0121(−10.96, −5.12)	− 0.044(−5.67, − 0.10)	0.043
Educational level			
Could not read & write	–	–	–
Primary	0.130(3.53, 7.14)	0.062(0.75, 4.31)	0.005
Secondary and above	0.110(4.78, 11.21)	0.067(1.84, 7.99)	0.002
Current job			
Farmer	–	–	–
Daily laborer	−0.076(−13,06, −3.42)	− 0.078(− 12.91, −4.10)	< 0.001
Employer	0.023(− 4.71, 15.14)	0.055(2.58, 17.59)	< 0.001
Family size	− 0.332(− 0.79, 0.13)	−0.201(−2.55, − 1.63)	< 0.001
Wealth Index	− 0.382(−8.26, −6.66)	−0.399 (− 8.67, − 6.90)	< 0.001
CBHI condition			
Un-insured	–	–	–
Insured	0.105(2.38, 5.82)	0.117 (2.98, 6.16)	< 0.001

## Discussion

In this study it was revealed that community based health insurance impacted the health-related quality of life among insured and un-insured family heads. Quality of life among insured household heads were higher compared with un-insured household heads; more specifically insured family heads had higher quality of life on the domain psychological and environmental health than un-insured family heads whereas low on physical health and almost equal on social relationship domain.

Quality of life scores in our study participants living separately by their marital condition were low compared with living together. This finding was consistent with reports conducted elsewhere [11]. The score represents the effect of living alone lead to deterioration of life which could be explained due to burden on taking family responsibility alone than joint care [12]. On the other hand, quality of life was markedly increased as an educational level increased. This result was consistent with the study finding conducted in nine European countries [13–15].

In addition, Study participants who engaged on daily labor during the study period negatively affected the quality of life. In contrary employed participants were positively associated for better quality of life as compared to farmers by their occupational status. This finding was similar with research report from Chinese and others [16, 17]. This effect may be explained due to job security and level of satisfaction on their job. Furthermore, the study revealed that the effect of family size and wealth index increment worth’s family head’s quality of life and it could be explained due to increased family responsibility.

### Limitation of the study

The major limitation of this study was related to causal relationship as it was not allowed to established cause and effect relationship. Except for gross socio-demographic character such as age, marital condition, educational status and job, this study did not allow to link with other potential factors that may affect quality of life. Despite these limitations, our study provided a comprehensive opportunity to overview the effect of health insurance on health related quality of life.

## Conclusions

From this work, we revealed that the two populations were different in their quality of life. Moreover, being member of community based health insurance had positive effect on health related quality of life. Triggered this, the government shall expand the community based health insurance into additional districts of Ethiopia and further actions should be established on the scheme satisfaction to ensure its continuity.

## Abbreviations

CBHI: Community Based Health Insurance
HRQoL: Health Related Quality of Life
IRB: Institutional Review Board
QOL: Quality of Life
SNNPR: South Nation Nationality People’s Region
SPSS: Statistical Package for Social Science
US$: United State Dollar
WHOQoL-BREF: World Health Organization Quality of Life Biomedical Research and Educational Facility
